# Notes and News

**Published:** 1894-01-13

**Authors:** 


### NOTES AND NEWS.

COLLECTED AND COMMUNICATED.

MR. HAYES, for ten years secretary to a private lunatic asylum near London, has, we understand, been appointed Secretary to the East London Hospital for Sick Children out of nearly 500 candidates.

THE long-disputed question of the fever hospital at Richmond is now settled. The Richmond Town Council are about to purchase seventeen acres of land on the southern corner of Hounslow Heath, separated from the Heath by the railway. The cost of the hospital is to be £6,000. The hospital belonging to the Heston and Isleworth Local Board at Dodswell is to be taken over by both authorities and used for small-pox cases. The hospitals will be situated about four miles from Richmond.

A MOST satisfactory announcement was made at the last meeting of the Metropolitan Asylums Board. This was that the accommodation for fever patients is sufficient for the needs of the present time. This, of course, indicates a great diminution in the spread of scarlet fever. The opposition to most sites chosen for new hospitals on the part of the Board is said to arise from the extreme costliness of the sites fixed upon. The last proposal is Shooter's Hill, where the cost of erection would be £446 per bed.

DR. JOHN A. WALLIS, the able Superintendent of the Lancaster County Asylum, Whittingham, has been appointed a Lunacy Commissioner for England. Dr. Wallis holds sound views on many questions, he is young enough to be vigorous and energetic, and we regard his appointment as full of promise for the improvement of many things which have failed to interest the older Commissioners, to the great regret of all who have a competent knowledge of the actual condition of lunacy matters in England to-day.

MR. CHAMBERLAIN states that the Royal Commission on Old Age Pensions is practically placed on the shelf for the present. He also expresses his opinion that the State should help support those amongst the poor who, having reached the age of 60 years, are after a life of toil unable to provide for themselves. This he thinks could be done through the agency of existing benevolent institutions.

IN spite of a certain prevalence of influenza in the metropolis, the death-rate last recorded from the Registrar General's office shows a rapid decrease. In fact, the deaths were below the average of the last ten years, and it may surprise those who, fearing London fogs, have flown to the health-giving air of Brighton, to learn that whilst London's death-rate was 23·3 per thousand of the inhabitants, that of Brighton was 27·0.

THE programme of the next Congress of Hygiene and Demography, which meet at Buda Pesth, is now shaping itself. The Congress is to open on September 2nd, and close on the 9th. It appears that the subject of diphtheria will occupy much attention. The English Committee was appointed as far back as 1891 at the Congress in London, but of this committee no chairman has yet been selected. It is proposed to organise an excursion to Constantinople after the Congress, and the inhabitants of Belgrade are also desirous of a visit from those attending the Congress.

WE notice that our contemporary *Truth* has buried the hatchet, so far as the St. John's Hospital for Skin Diseases is concerned, in an article which is most notable for the measured meed of praise it contains. From the outset of this controversy we have maintained that until a public inquiry was held which would afford full opportunity for an impartial investigation of the many charges of abuses brought against the St. John's Hospital for Skin Diseases, careful contributors must necessarily prefer to give their money to hospitals where the management was less suspect and more satisfactory. *Truth* seems to have confined the inquiries made to the business management, but the worst abuses of all were alleged to affect the conduct of the out and in patients departments, and the personnel which Mr. Labouchere's inquiries do not seem to have touched.

A GENERAL committee is being formed by friends of the late Sir Andrew Clark, to take steps to found a memorial to his name, in recognition of his distinguished professional career. The first meeting of this committee was held Thursday, the 11th inst. It is generally felt most suitable that the London Hospital should participate in any memorial scheme in memory of its late honoured physician, as to it he did not fail to attribute much of the experience and skill through which he benefited mankind. It is, therefore, proposed that the memorial should consist of a new wing, to be added to the London Hospital, bearing the name of Sir Andrew Clark. The Duke of Cambridge has consented to act as Chairman of the Fund Committee; and Mr. Gladstone, signifying his hearty sympathy with the movement, has contributed £100 towards it.

